# Detection of circulating prostate-specific antigen-positive cells in patients with prostate cancer by flow cytometry and reverse transcription polymerase chain reaction.

**DOI:** 10.1038/bjc.1996.372

**Published:** 1996-08

**Authors:** E. J. Fadlon, R. C. Rees, C. McIntyre, R. M. Sharrard, J. Lawry, F. C. Hamdy

**Affiliations:** Institute for Cancer Studies, University of Sheffield Medical School, UK.

## Abstract

**Images:**


British Journal of Cancer (1996) 74, 400–405
© 1996 Stockton Press All rights reserved 0007-0920/96 $12.00

### Detection of circulating prostate-specific antigen-positive cells in patients with prostate cancer by flow cytometry and reverse transcription polymerase chain reaction

EJ Fadlon[1], RC Rees[1], C McIntyre[1], RM Sharrard[1], J Lawry[1] and FC Hamdy[2]

[1]*Institute for Cancer Studies, University of Sheffield Medical School, Sheffield S10 2RX;* [2]*University Urology Unit, Freeman Hospital, Newcastle upon Tyne, NE7 7DN, UK.*

**Summary** The presence of prostate-specific antigen (PSA)-positive cells has previously been demonstrated in the peripheral blood of prostate cancer patients by flow cytometry (FC), but the identity of these cells has not been established. In this study, the reverse transcriptase polymerase chain reaction (RT–PCR) was compared with analytical FC in an attempt to detect and characterise these cells. Peripheral blood was obtained from 12 patients with newly diagnosed and untreated prostate cancer and five controls. Nine of the 12 patients with prostate cancer (75%) had circulating PSA-positive cells as shown by FC. Only one of those patients (11.1%) was found to express PSA mRNA by RT–PCR. The absence of PSA mRNA in the majority of samples showing PSA-positive cells suggests that they do not represent haematogenous micrometastases. PSA-positive cells in the blood could represent monocytes that express PSA, either following binding/phagocytosis of free serum PSA or phagocytosis of tumour cells.

**Keywords:** prostate cancer; prostate-specific antigen; micrometastases; reverse transcriptase polymerase chain reaction; flow cytometry

Prostate cancer is the third most common malignancy in men in England and Wales, with over 10 000 new cases and 8000 deaths from the disease every year (OPCS, 1993). Over half the patients present with locally advanced and/or metastatic disease, and can be treated by palliative measures only. In addition, once early tumours are detected, prognosis is largely unpredictable by current investigative methods. Clinicians are unable to predict disease progression and to inform the patient whether his tumour is likely to progress, or whether any form of treatment will alter the outcome. New criteria to define the aggressive and metastatic potential of early prostate cancer are needed, particularly in view of the recent controversies and evidence from North American studies questioning the benefits of radical surgery over observation in early stage prostatic adenocarcinoma (Fleming *et al.*, 1993; Lu-Yao *et al.*, 1993; Chodak *et al.*, 1994). Furthermore, even in cases where the disease appears to be confined to the prostate, cancers are understaged in over 50% of cases, with resulting positive surgical margins, extracapsular extension and potential treatment failure (Epstein *et al.*, 1993).

The formation of metastasis is a significant, rate-determining event in the progression of cancer. It is a complex, non-random phenomenon involving a cascade of multisequential events, including tumour cell detachment from the primary lesion into the blood and lymphatic channels, survival of a selected population of malignant cells in a hostile environment, extravasation at a chosen site and the final formation of a secondary deposit (Poste and Fidler, 1980).

In an attempt to isolate circulating tumour cells in prostate cancer patients before their actual deposition at a distant site and metastasis formation, we have previously demonstrated the presence of circulating prostate-specific antigen (PSA)-positive cells using monoclonal antibody (MAb) staining for PSA and flow cytometric analysis (Hamdy *et al.*, 1992). Quantification of circulating PSA-positive cells appeared to be a more sensitive predictor of bone scan findings than serum PSA estimation. The phenotype of these circulating PSA-positive cells however, remains unclear, particularly in view of the large percentage of these cells found in peripheral mononuclear cell suspensions (up to 50% of separated cells). Recent studies have demonstrated that haematogenous micrometastases can be detected in some but not all patients with different stages of prostate cancer using a reverse transcription polymerase chain reaction (RT–PCR) based technique (Moreno *et al.*, 1992; Katz *et al.*, 1994; Israeli *et al.*, 1994). One study suggested that 'molecular staging' of prostate cancer patients by RT–PCR is superior to all other available staging methods (Katz *et al.*, 1994). Using a similar protocol, involving reverse transcriptase PCR (RT–PCR) and Southern blotting, we have compared cytometric analysis of PSA-positive circulating cells with RT–PCR in an attempt to determine whether the peripheral circulating PSA-positive cells represent a tumour cell population. The results and their implications are discussed.

### Patients, materials and methods

### *Patients*

Twelve patients with histologically proven and untreated carcinoma of the prostate (CaP) were studied. Three men with benign prostatic hyperplasia and two healthy females were used as controls. All men had three serial serum prostate-specific antigen measurements (immunoradiometric assay, CIS, UK) before prostatic manipulation, and patients with CaP were staged by digital rectal examination, transrectal ultrasonography, and technetium 99 m isotope bone scanning. Six patients (50%) had evidence of skeletal metastases as shown by a positive isotope bone scan. Two patients had apparently localised disease, and the remaining four had locally advanced tumours. Prostatic biopsies were obtained transrectally and diagnosis was made by standard histopathological criteria. All men with evidence of significant symptomatic bladder outflow obstruction were treated by transurethral resection of the prostate, and the tissue obtained was histologically examined further. Patients with metastatic CaP were treated by hormonal manipulation either in the form of bilateral subcapsular orchidectomy or

Correspondence: FC Hamdy
Received 17 November 1995; revised 2 February 1996; accepted 22 February 1996

administration of a luteinising hormone-releasing hormone (LHRH) analogue. Men with tumours confined to the prostate or locally advanced disease were treated by external beam irradiation. Follow-up ranged from 10 to 21 months (median 19 months). Of the six patients with metastases, one had aggressive hormone refractory disease and died within one week of inclusion in the study. All the other men were alive and well at the last follow-up, having responded to the various treatments administered. Patients' details are summarised in Table I.

*Sample preparation*

*Prostatic tissue* Prostatic tissue was obtained from transrectal core biopsies and resection specimens before definitive therapy and histologically examined. Tissue samples were collected in sterile cryovials, placed on ice and stored at −80°C until processed. Specimens were minced into 1 mm cubes with crossed scalpels then used for RNA extraction. Benign prostatic hyperplastic (BPH) tissue was used as PSA-positive control.

*Prostate cancer cell line* The prostatic cancer cell line LNCaP (kindly provided by Dr M Harper, Tenovus Institute, Cardiff, UK) was also used as a positive control for PSA-positive cells. The cell line was grown in RPMI-1640 medium (Gibco, Paisley, UK) supplemented with 10% heat-inactivated fetal calf serum (HIFCS) (Northumbria Biologicals, Cramlington, UK) and cells were stored as a pellet of $10^7$ cells at −80°C until processed.

*Blood samples* Ten millilitres of peripheral venous blood was collected in EDTA vacutainers from male patients before treatment, and from healthy female volunteers. Peripheral blood mononuclear cells (PBMCs) were isolated by density gradient centrifugation (J Prep, TechGen International, London, UK) and subsequently tested for antigen expression and ploidy status using flow cytometry. Cells used for RNA extraction were stored as a pellet of $10^7$ cells at −80°C until processed.

*MAb staining and flow cytometry*

Approximately $10^6$ PBMCs were stained with MAbs against PSA and leucocyte common antigen (LCA) which was used as a control (Dakopatts UK) by the two-stage method in conjunction with fluorescein isothyocyanate (FITC)-conjugated goat anti-mouse f(ab')$_2$ antibody (Caltag, Bradshaw Biologicals, Leics, UK) ($1 \times 10^6$ PBMC pellet). FITC-conjugated goat anti-mouse negative control was included. DNA ploidy status was measured by flow cytometry (Ortho Diagnostic Orthocyte), using propidium iodide (DNA CycleTest, Becton-Dickinson, Cowley, UK) and at least 10 000 cells analysed.

*Oligonucleotide primers and probes*

Oligonucleotide primers and probes were designed and checked for specificity using the SEQNET facility (SERC, Daresbury Laboratory, UK). The sequences used were as follows:

Antisense PCR primer (exon 3):
5'-ACTCCTCTGGTTCAATGCTG-3'
Sense PCR primer (exon 2):
5'-TCATCCTGTCTCGGATTGTC-3'
Exon 3 probe:
5'-CCGACCCAGCAAGATCACGC-3'

These primers resulted in the amplification of a 426 basepair PCR product. Primers were synthesised by R & D Systems (Europe), and the probe was synthesised in the department using an Applied Biosystems 491 PCR-Mate.

There is a high homology between PSA and the human kallikrein (HMGK) genes (Riegmann *et al.*, 1989). The primers selected were specific for PSA and did not cross-react with HMGK. Primer and magnesium concentrations were titrated in order to avoid false-positives due to random products. Both LNCaP and BPH tissues were used as the positive controls for PSA expression. The amount of RNA (1–5 μg) was titrated for positive controls, both 1 and 5 μg of PBMC sample RNA were added to the reverse transcriptase (RT) mix; at least one RNA-free control was included for the RT procedure and at least two cDNA-free controls for the PCR.

*Labelling of oligonucleotide probes*

Oligonucleotide probe (1 μg) was end-labelled with digoxigenin dUTP by incubation at 37°C for 3 h in a 50 μl reaction volume consisting of 1 × terminal deoxynucleotidyl transferase (TdT) buffer (Life Technologies, Paisley, UK), 5 μM digoxigenin dUTP (Boehringer Mannheim, Lewes, UK), 30 mM Tris HCl pH 6.8, 10 units of inorganic pyrophosphatase (Sigma Chemical Co., Poole, UK) and 50 units of TdT (Life Technologies). The labelled oligonucleotide was then stored at −20°C until use.

**Table I** Summary of patients' details

| Patients | | Staging (TNM) | Grading (Gleason score) | sePSA (ng ml$^{-1}$) | PSA-positive cells (%) | Southern blot | Treatment |
|---|---|---|---|---|---|---|---|
| 1. | CaP | T3NxM0 | 6 | 81 | 9 | Negative | TURP + DXT |
| 2. | CaP | T3NxM1 | 7 | 87 | 10 | Negative | TURP + HM |
| 3. | CaP | T4NxM1 | 9 | > 120 | 1.5 | Negative | TURP + HM |
| 4. | CaP | T4NxM1 | 8 | 117 | 13 | Negative | HM |
| 5. | CaP | T3NxM0 | 5 | 32 | 2 | Negative | TURP + DXT |
| 6. | CaP | T2bNxM0 | 6 | 31 | 0 | Negative | DXT |
| 7. | CaP | T3NxM0 | 8 | 35 | 3 | Negative | TURP + DXT |
| 8. | CaP | T4NxM1 | 9 | > 120 | 2 | Positive | TURP + HM |
| 9. | CaP | T4NxM1 | 8 | > 120 | 7 | Negative | DXT |
| 10. | CaP | T2bNxM0 | 6 | 27 | 0 | Negative | DXT |
| 11. | CaP | T3NxM0 | 9 | 83 | 2 | Negative | TURP + HM |
| 12. | CaP | T4NxM1 | 7 | > 120 | 0 | Negative | TURP + HM |
| 13. | BPH | – | – | 5 | 0 | Negative | TURP |
| 14. | BPH | – | – | 9 | 0 | Negative | TURP |
| 15. | BPH | – | – | 1.4 | 0 | Negative | TURP |
| 16. | HF | – | – | 0.1 | 0 | Negative | |
| 17. | HF | – | – | 0.1 | 0 | Negative | |

CaP, carcinoma of the prostate; BPH, benign prostatic hyperplasia; HF, healthy female; sePSA, serum prostate-specific antigen (120 ng ml$^{-1}$, maximum measurement given by laboratory); TURP, transuretheral resection of the prostate; HM, hormone manipulation; DXT, deep X-ray therapy.

*Extraction of RNA*

All solutions were pretreated with diethylpyrocarbonate (DEPC) (Sigma) or made up with DEPC-treated water, then autoclaved, and all glassware baked at 250°C for 4 h, to ensure that the solutions were RNAase free. Total RNA was extracted from $10^7$ PBMCs by modification of the method of Chomczynski and Saachi (1987) using an RNAzol B RNA extraction kit (Biogenesis, Bournemouth, UK). The RNA pellet was resuspended in 100 $\mu$l of 0.5 mM EDTA then precipitated overnight at −20°C in the presence of 50 $\mu$l 3 M sodium acetate and 400 $\mu$l of absolute ethanol. The resulting precipitate was washed once in 75% ethanol and air dried. RNA was resuspended in 21 $\mu$l of 0.5 mM EDTA. The optical density of a 1 in 100 dilution was determined at 260 nm and 280 nm.

*cDNA synthesis*

Either 1 $\mu$g or 5 $\mu$g of RNA was adjusted to a final volume of 22 $\mu$l using DEPC-treated water and 28 $\mu$l of reverse transcriptase reaction mixture was added. This reaction mixture was composed of 100 mM Tris HCl pH 8.3, 150 mM potassium chloride, 6 mM magnesium chloride, 12 mM dithiothreitol, 10 $\mu$g random primer pd(N)$_6$ (Pharmacia, Milton Keynes, UK), 0.6 mM dNTPs (Pharmacia), and 400 units M-MLV reverse transcriptase (Life Technologies). The reaction mixture was then incubated at 37°C for 1h after which the samples were used immediately or stored at −20°C.

*Polymerase chain reaction (PCR)*

Five microlitres of cDNA preparation was added to the PCR reaction mixture in a final volume of 50 $\mu$l containing the following: 1 × *Taq* DNA polymerase incubation buffer (Boehringer Mannheim), 0.2 mM each dATP, dCTP, dTTP and dGTP (Pharmacia), 100 pmol of each oligonucleotide primer and 1 unit *Taq* DNA polymerase (Pharmacia). The mixture was overlaid with 50 $\mu$l of mineral oil (Sigma) and the amplification performed in a Perkin Elmer cycler as follows: 2 min at 95°C; 35 cycles of 30 s at 95°C; 30 s at 55°C; 1 min 30 s at 72°C (increasing by 6 s per cycle); followed by a final 15 min extension period at 72°C. The reaction mixture was then stored at 4°C until analysis. cDNA-free negative controls were included for each RT−PCR.

A single cell suspension of BPH tissue was prepared by mincing with crossed scalpels followed by filtration through a fine mesh. BPH cells were added to PBMCs obtained from healthy females at the following ratios of BPH:PBMC: 1:$10^2$, 1:$10^3$, 1:$10^4$, 1:$10^5$, 1:$10^6$. The PBH:PBMC cell suspension was centrifuged and the supernatant discarded. RNA was extracted from PBH-spiked PBMCs in order to determine the sensitivity of the RT−PCR under the selected assay conditions.

*Analysis of PCR products*

Chloroform (100 $\mu$l) and 13 $\mu$l of sample buffer (0.1% bromophenol blue, 50% glycerol) were added to each tube which was then shaken and centrifuged for 1 min at 7000 ***g***. Twenty-five millilitres of the upper layer was loaded onto a 1.5% agarose gel (Sea Kern GTG, Flowgen) containing TAE buffer (40 mM Tris acetate, pH 8.3, 1 mM EDTA) immersed in TAE running buffer. A 123 bp ladder (Life Technologies) and digoxigenin-labelled markers VI (Boehringer Mannheim) were included on each gel. Electrophoresis was performed at 100 V for 3 h, stained with 0.5 $\mu$g ml$^{-1}$ ethidium bromide for 30 min then examined under a UV light. The gel was washed twice, for 10 min, in 0.4 M sodium hydroxide then blotted overnight onto positively charged nylon membrane (Boehringer Mannheim). The blots were washed twice, for 10 min, in 2 × TSB (1 × TSB = 150 mM sodium chloride, 15 mM Tris HCl, pH 7.5), air dried and baked at 95°C for 40 min.

Blots were prehybridised at 55°C for at least 2 h in a prehybridisation solution [4 × TSB, 0.1% Tween 20 (Sigma), 1% blocking reagent (Boehringer Mannheim), 100 $\mu$g ml$^{-1}$ sonicated salmon sperm DNA (Sigma), pH 7.5]. Digoxigenin-labelled oligonucleotide probe was added to the prehybridisation solution at a final concentration of 2 ng ml$^{-1}$ and hybridisation performed at 55°C for 16 h. The blots were washed in decreasing TSB concentrations (4 × TSB to 0.1 × TSB), at room temperature, 15 min per wash, and again for 30 min, at room temperature, in a blocking solution [1 × TBS (150 mM sodium chloride, 100 mM Tris HCl, pH 7.5), 0.1% Tween 20, 1% blocking reagent]. Then 3.75 units of alkaline phosphatase-conjugated sheep anti-digoxigenin IgG Fab fragment (Boehringer Mannheim) were added and the incubation continued for a further 30 min. Unbound antibody was removed by two 15 min washes in 1 × TBS followed by a 5 min equilibration in substrate buffer (100 mM sodium chloride, 100 mM Tris HCl, pH 9.5). The membrane was then soaked in substrate buffer containing 0.1 mg ml$^{-1}$ Lumigen PPD (Boehringer Mannheim), placed between two acetate sheets and exposed to preflashed Hyperfilm MP (Amersham, Aylesbury, UK) at 37°C for 10−30 min.

### Results

*Flow cytometry, DNA and scatter analysis*

Nine out of 12 patients with prostate cancer (75%) had circulating PSA-positive cells ranging from 1.5−13% of isolated PBMC populations as detected by FC. DNA analysis of PBMCs showed all cells to be diploid. Patient 8 (Table I) showed an unusual scatter distribution for PBMCs, and a cell population was detected of similar or greater size to the monocyte population by normal scatter analysis (Figure 1).

*Detection of PSA mRNA by RT−PCR*

Following the BPH spiking experiment under the conditions outlined, we were able to detect one PSA-positive cell per $10^4$ PBMCs by RT−PCR (two titrations, cDNA samples run at least twice). The RT−PCR was 100-fold more sensitive than the flow cytometric analysis threshold of one PSA-positive cell per 100 PBMCs. Positive controls (BPH tissue) and PBMC samples from negative controls and patients were reverse transcribed and subjected to PCR amplification using PSA-specific primer sequences. Following gel electrophoresis and ethidium bromide staining, a PCR product corresponding to a 426 bp fragment was detectable in all positive controls, but not in the negative controls or patient PBMC samples. For more sensitive detection, the gels were blotted and hybridised with the antisense oligonucleotide probe corresponding to a unique sequence of exon 3 (see Materials and methods). This allowed the detection of a band of 426 bp in a single patient sample (Figure 2b). This sample was obtained from patient 8 (Table I) who had an aggressive, poorly differentiated locally advanced and metastatic tumour (T4NxM1 Gleason score 9) which did not respond to hormone manipulation, and also demonstrated an unusual PBMC scatter analysis (illustrated in Figure 1a). RT−PCR for LNCaP cells was carried out as a second positive control with 1−5 $\mu$g of total RNA. Products were detected for all RNA concentrations. LNCaP cells and tissue extracts were shown to express up to three bands; controls for PBMCs, cDNA synthesis, PCR and RNAzol were all negative. Figure 2a shows negative (female PBMCs), positive controls (LNCaP) and negative patients.

### Discussion

The detection of circulating tumour cells in cancer patients is not by any means a new phenomenon. Indeed, several

**Figure 1** (**a**) Scatter analysis of samples prepared by density gradient centrifugation. Lymphocytes and monocytes were identified by conventional forward (FSC) and side (SSC) scatter with contaminating granulocytes at less than 3% of cells (A) compared with 23% in B. (**b**) Dual parameter displays show MAb fluorescence (FITC) *vs* side scatter for the isotype control (C), PSA-stained cells (D) and LCA-stained cells (E).

previous studies, some dating back over half a century, have elegantly demonstrated the presence of circulating malignant cells in the peripheral blood of patients with advanced disease (Warren and Gates, 1936; Engell, 1955; Fidler, 1970; Schwartz *et al.*, 1995), without the help of current sophisticated and molecular biology techniques. Previous studies in animals have shown that metastasis does not rely on the random survival of cells released from the primary tumour, but from the selective growth of specialised subpopulations of highly metastatic cells endowed with properties which will allow them successfully to complete each step of the metastatic cascade (Fidler, 1970; Fidler and Kripke, 1977). Based on these principles of tumour metastasis, it has been our intention to isolate circulating tumour cells from patients with clinically undetectable metastases for two purposes: firstly, to identify a group of patients with metastatic potential before the firm establishment of secondary deposits, thus allowing early aggressive treatment, and secondly, to isolate the selected survivor tumour cell escaping into the circulation in order to compare its biological and genetic properties with cells from the primary tumour. Analytical FC and tissue-specific MAb staining have been used successfully to detect circulating PSA-positive cells in patients with prostate cancer. However, some doubt arose as to whether the cells were solely of prostatic origin (Hamdy *et al.*, 1992), and the purpose of the present study was to identify the phenotype of circulating PSA-positive cells, confirming or excluding their prostatic origin by detecting mRNA expression for PSA. Moreno *et al.* (1992) in a similar study, have demonstrated the ability of RT–PCR to detect haematogenous micrometastases in patients with advanced prostate cancer, but failed to detect any of these cells in patients with clinically non-metastatic disease. Katz *et al.* (1994), also using RT–PCR, have been able to detect circulating prostate cells in patients with apparently localised disease undergoing radical prostatectomy, and found a strong correlation between a positive PCR reaction, capsular tumour penetration and positive surgical margins, suggesting the potential of this technique to be used for 'molecular staging' of prostate cancer. Two further studies by Israeli *et al.* (1994, 1995) used nested RT–PCR to compare the sensitivity of PSA with prostate-specific membrane antigen (PSMA) in the detection of circulating prostatic cells. The studies showed that PSMA was significantly more sensitive than PSA, but the group could not reproduce the results reported by Katz *et al.* (1994) in that they failed to detect with any significance the presence of micrometastatic prostatic cells in patients with pathologically organ-confined disease. In turn, Cama *et al.* (1995) repeated the initial experiments made by Katz *et al.* (1994) using the same patients' samples, comparing PSA with PSMA, and found, in contrast with Israeli *et al.* (1995) that PSA was more sensitive than PSMA in predicting local tumour penetration, adding further controversy to the possible value of these sensitive assays in staging prosate cancer. It is interesting to

**Figure 2** (**a**) Southern blotting depicting detection of PSA. Lane 1, Molecular weight markers; lane 2, female PBMC negative control; lanes 3 and 4, LNCaP cell line positive control; lanes 5–8, RT–PCR-negative patients; lanes 9–11, patients with benign prostatic hyperplasia (BPH). (**b**) Southern blotting depicting detection of PSA. Lane 1, molecular weight markers; lanes 2–4, RT–PCR-negative patients; lane 5, RT–PCR-positive patient; lane 6, RNA control; lane 7, cDNA control; lane 8, PCR control; lanes 9–11, BPH tissue-positive control.

note that the authors of all these studies assume that circulating PSA-positive cells are endowed with metastatic propensity, despite the fact that the results only demonstrate the presence of cells of prostatic origin. Furthermore, the high sensitivity of some assays using the nested primer PCR technique, resulting in an improved detection rate of prostatic cells from one per 10 000 to one per million cells may interfere with the ability of these tests to identify cells of genuine prostatic origin. The specificity of PSA mRNA in detecting true prostatic cells has been questioned in a recent study by Smith *et al.* (1995). Using the nested primer PCR method, the authors demonstrated the presence of PSA mRNA in non-prostatic cell lines, including ovarian, lung, myeloid leukaemia and normal blood; an important observation which must be taken into consideration when interpreting results generated by these sensitive methods. In our positive controls, we observed expression of two extra bands by both LNCaP cells and tissue extracts, as illustrated in Figure 1. These bands remain to be identified, but one could speculate that they may represent spliced variants of PSA. The present study did not attempt to verify the validity of the PSA RT–PCR assay nor its sensitivity and specificity in the detection of extracapsular prostate cancer. Should further studies confirm the malignant nature of these circulating cells, more extensive work would be required to assess their metastatic capability. Although our study failed to characterise the phenotype of circulating PSA-positive cells detected by FC, and despite the small number of patients investigated, our findings whereby PSA mRNA was detected in only one of nine men (11.1%) with prostate cancer and circulating PSA-positive cells, supports our initial hypothesis that the majority of these cells are not of prostatic origin, but represent a population of cells which express PSA at the cell surface. Whether circulating PSA-positive cells are mononuclear phagocytes which have phagocytosed circulating tumour cells and re-expressed PSA, or whether PSA has passively adhered to the surface of these immunocytes remains to be established. This hypothesis warrants clarification by further experimentation into the cellular origin of the non-prostatic PSA-positive cells in the blood stream of prostate cancer patients. Studies we are currently undertaking may reveal novel mechanisms of PSA handling by the immune system, and allow us to improve our understanding of the biology of prostate cancer.

**Acknowledgements**
This study was supported by the trustees for the former United Sheffield Hospitals, the Yorkshire Cancer Research Campaign, the North of England Cancer Research Campaign and benefited from the use of the SEQNET facility.

### References

CAMA C, OLSSON CA, RAFFO AJ, PERLMAN H, BUTTYAN R, O'TOOLE K, MCMAHON D, BENSON MC AND KATZ AE. (1995). Molecular staging of prostate cancer. II. A comparison of the application of an enhanced reverse transcriptase polymerase chain reaction assay for prostate specific antigen verus prostate specific membrane antigen. **153,** 1373–1378.

CHODAK GW, THISTED RA, GERBER GS, JOHANSSON J-E, ADOLFSSON J, JONES GW, CHISHOLM GD, MOSKOVITZ B, LIVNE PM AND WARNER J. (1994). Outcome following conservative management of patients with clinically localized prostate cancer. *N. Eng. J. Med.*, **330,** 242–248.

CHOMCZYNSKI P AND SACCHI N. (1987). Single-step method of RNA isolation by acid guanidium thiocyanate–phenol–chloroform extraction. *Anal. Biochem.*, **162,** 156–159.

ENGELL HC. (1995). Cancer cells in the circulating blood. Clinical study on occurrence of cancer cells in peripheral blood and in venous blood draining tumour area at operation. *Acta Chir. Scand.* (suppl), **201,** 1–70.

EPSTEIN JI, CARMICHAEL MJ, PIZOV G AND WALSH PC. (1993). Influence of capsular penetration on progression following radical prostatectomy: a study of 196 cases with long-term follow-up. *J. Urol.*, **150,** 135–141.

FIDLER IJ. (1970). Metastasis: Quantitative analysis of distribution and fate of tumour emboli labelled with 125I-5-iodo-2'-deoxyuridine. *J. Natl Cancer Inst.*, **45,** 773–782.

FIDLER IJ AND KRIPKE ML. (1977). Metastasis results from pre-existing variant cells within a malignant tumor. *Science*, **197,** 893–895.

FLEMING C, WASSON JH, ALBERTSEN PC, BARRY MJ AND WENNBERG JE. FOR THE PROSTATE PATIENT OUTCOMES RESEARCH TEAM. (1993). A decision analysis of alternative treatment strategies for clinically localised prostate cancer. *JAMA*, **269,** 2650–2658.

HAMDY FC, LAWRY J, ANDERSON JB, PARSONS MA, REES RC AND WILLIAMS JL. (1992). Circulating prostate specific antigen-positive cells correlate with metastatic prostate cancer. *Br. J. Urol.*, **69,** 196–392.

ISRAELI RS, MILLER WH, SU SL, POWELL T, FAIR WR, SAMADI DS, HURYK RF, DEBLASIO A, EDWARDS ET, WISE GJ AND HESTON WDW. (1994). Sensitive nested reverse transcription polymerase chain reaction detection of circulating prostatic tumor cells: comparison of prostate-specific membrane antigen and prostate-specific antigen-based assays. *Cancer Res.*, **54,** 6306–6310.

ISRAELI RS, MILLER WH, SU SL, SAMEDI DS, POWELL CT, HESTON WDW AND FAIR WR. (1995). Sensitive detection of prostatic hematogenous tumor cell dissemination using prostate specific antigen and prostate specific membrane-derived primers in the polymerase chain reaction. *J. Urol.*, **153,** 573–577.

KATZ AE, OLSSON CA, RAFFO AJ, CAMA C, PERLMAN H, SEAMAN E, O'TOOLE KM, MCMAHON D, BENSON MC AND BUTTYAN R. (1994). Molecular staging of prostate cancer with the use of an enhanced reverse transcriptase–PCR assay. *Urology* **43,** 765–774.

LU-YAO GL, MCLERRAN D, WASSON J AND WENNBERG JE. FOR THE PROSTATE PATIENT OUTCOMES RESEARCH TEAM. (1993). An assessment of radical prostatectomy. *JAMA*, **269,** 2633–2636.

MORENO JG, CROCE CM, FISCHER R, MONNE M, VIHKO P, MULHOLLAND SG AND GOMELLA LG. (1992). Detection of hematogenous micrometastasis in patients with prostate cancer. *Cancer Res.*, **52,** 6110–6112.

OFFICE OF POPULATION, CENSUSES AND SURVEYS. (1993). *Cancer Statistics. Registrations 1987.* Series MB1 No. 20. HMSO: London.

POSTE G AND FIDLER IJ. (1980). The pathogenesis of cancer metastasis. *Nature*, **283,** 139–146.

RIEGMAN PHJ, VLIESTRA RJ, VAN DER KORPUT JAGM, ROMJIN JC AND TRAPMAN J. (1989). Characterization of the prostate-specific antigen gene: a novel human kallikrein-like gene. *Biochem. Biophys. Res. Commun.*, **159,** 95–102.

SCHWARTZ R, WALK A, TOOMES H AND SCHIRRMACHER V. (1985). Assay for the determination of human carcinoma cells in circulating blood. *J. Cancer Res. Clin. Oncol.*, **109,** 122–129.

SMITH MR, BIGGAR S AND HUSSAIN M. (1995). Prostate specific antigen messenger RNA is expressed in non-prostate cells: implications for detection of micrometastases. *Cancer Res.*, **55,** 2640–2644.

WARREN S AND GATES O. (1936). Fate of intravenously injected tumor cells. *Am. J. Cancer*, **27,** 485–492.


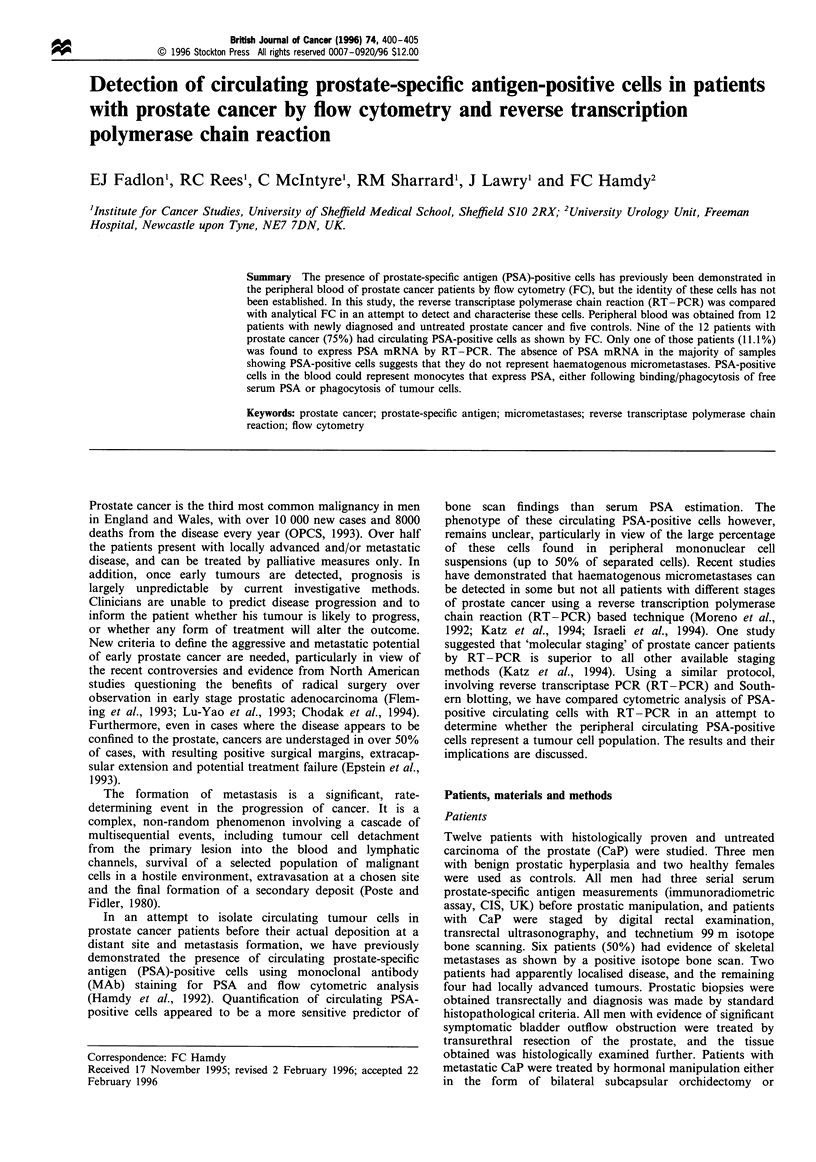

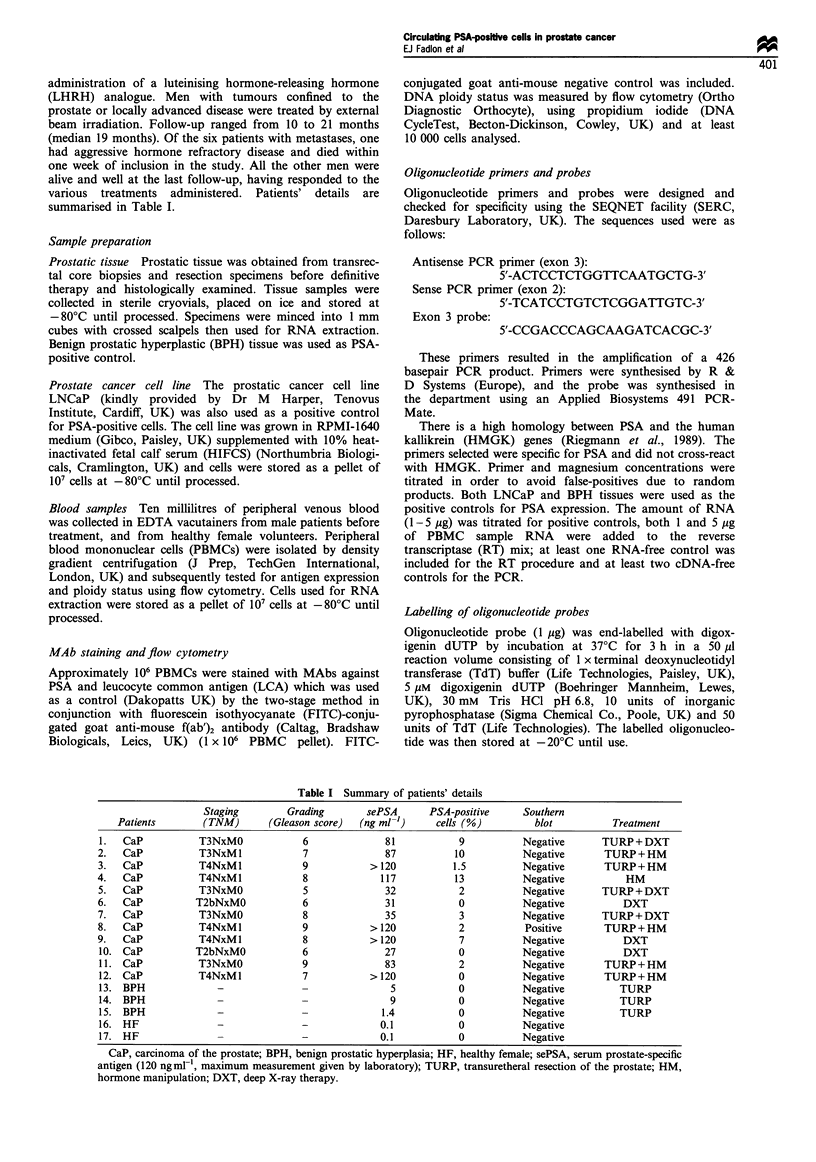

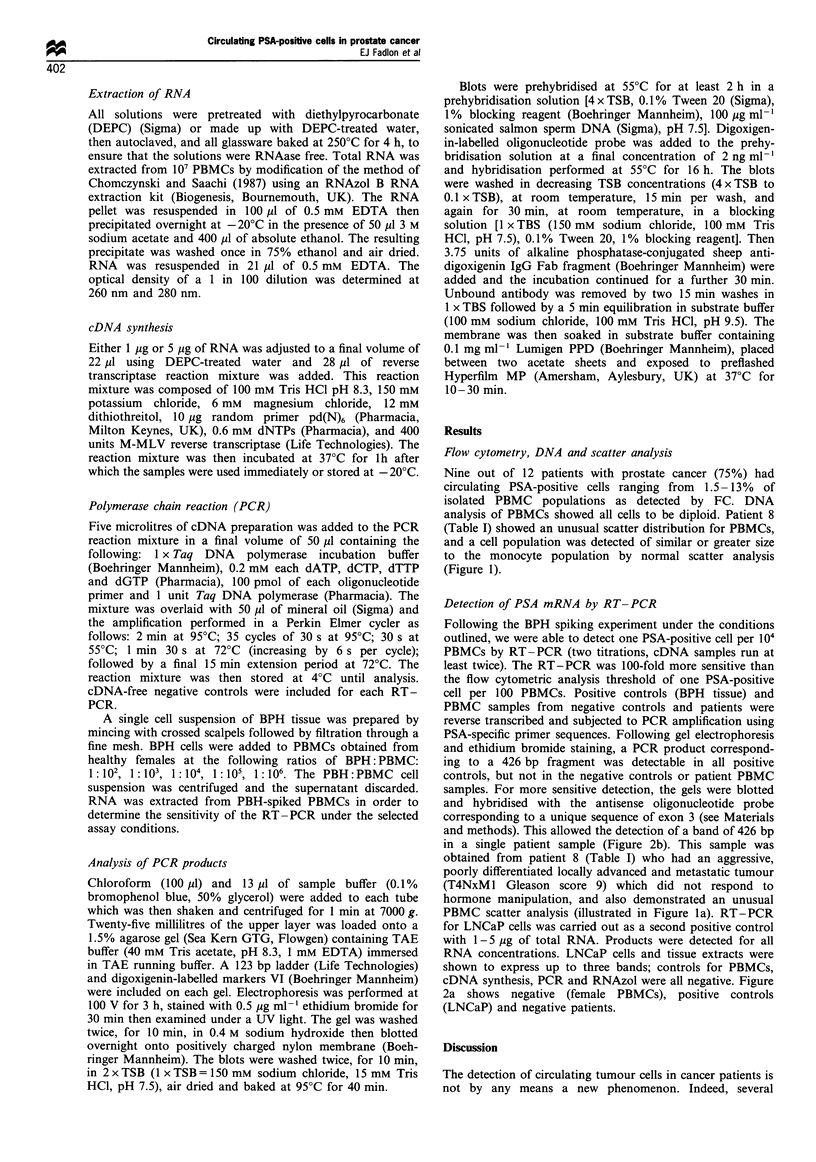

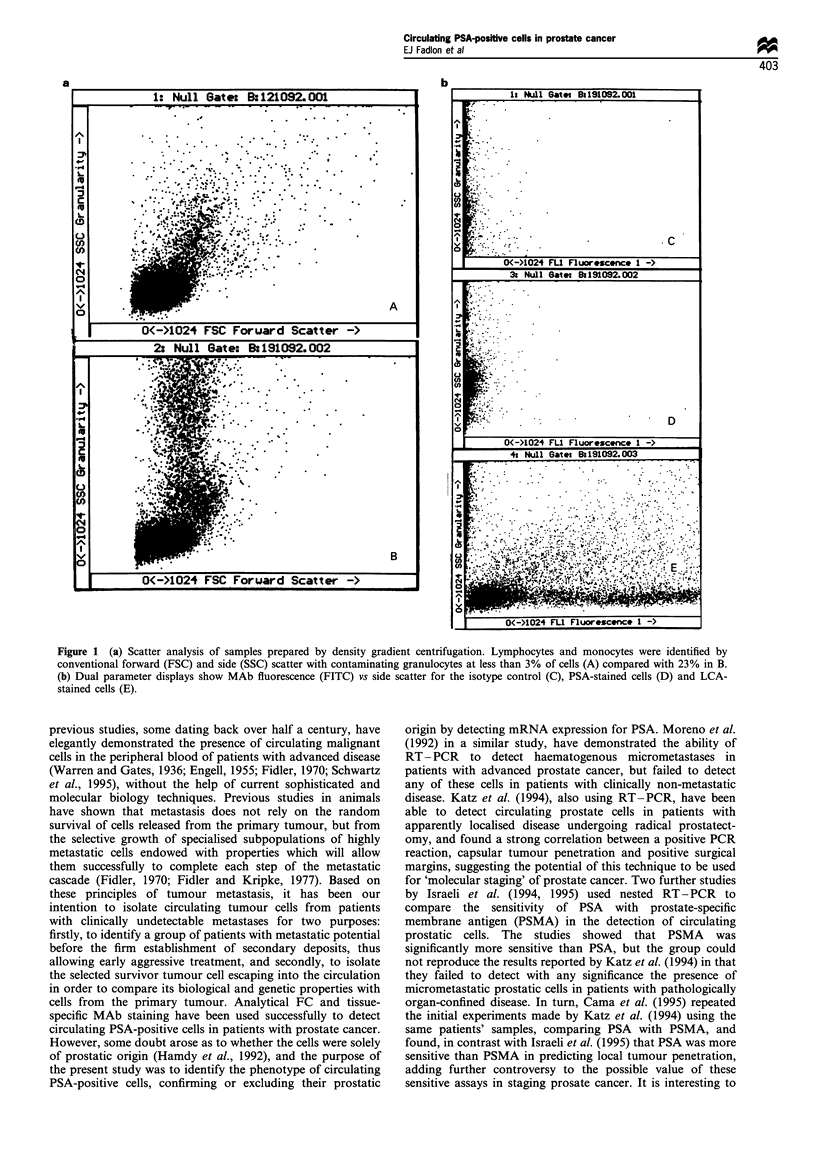

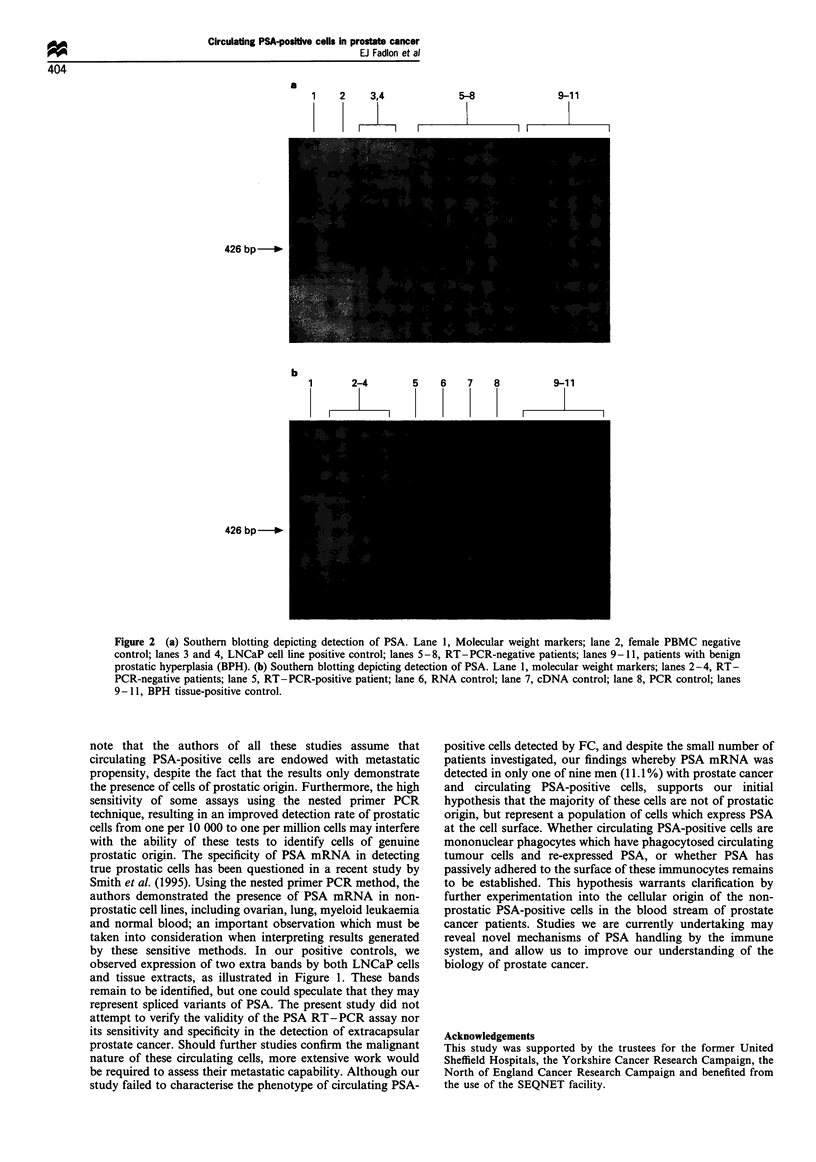

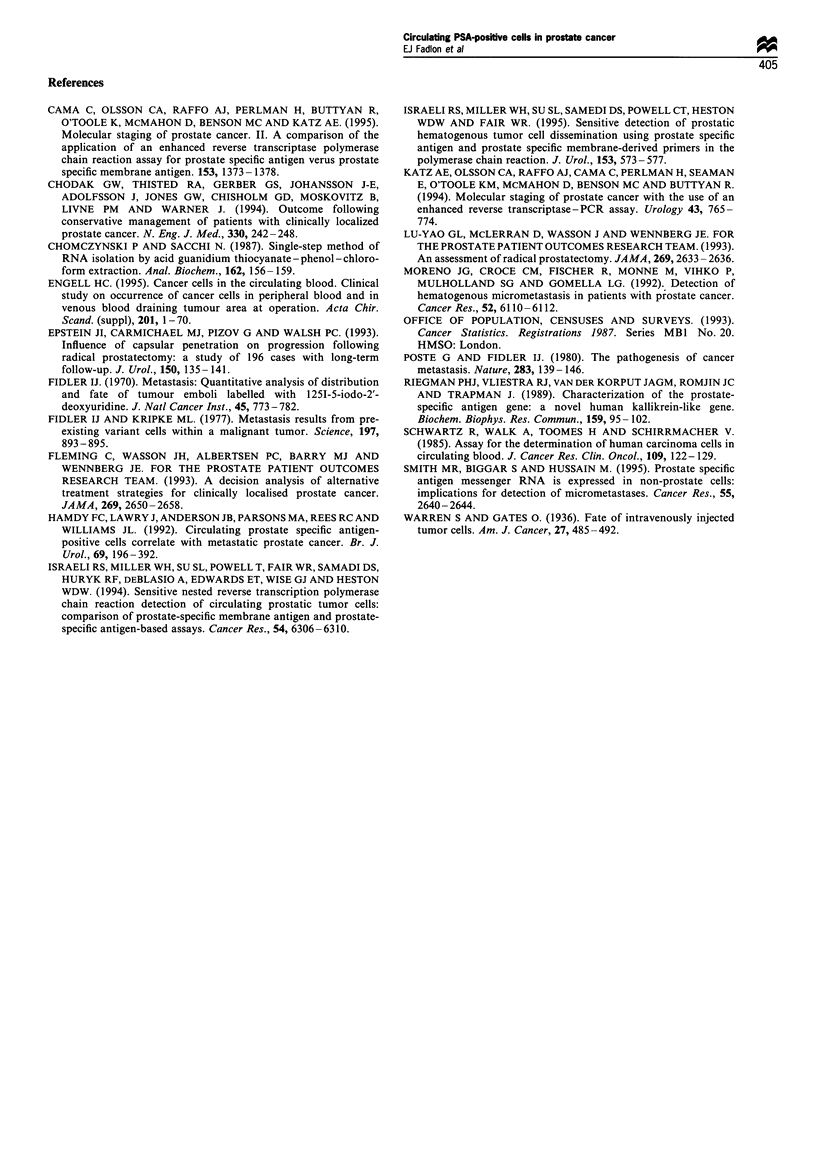


## References

[OCR_00737] Cama C., Olsson C. A., Raffo A. J., Perlman H., Buttyan R., O'Toole K., McMahon D., Benson M. C., Katz A. E. (1995). Molecular staging of prostate cancer. II. A comparison of the application of an enhanced reverse transcriptase polymerase chain reaction assay for prostate specific antigen versus prostate specific membrane antigen.. J Urol.

[OCR_00744] Chodak G. W., Thisted R. A., Gerber G. S., Johansson J. E., Adolfsson J., Jones G. W., Chisholm G. D., Moskovitz B., Livne P. M., Warner J. (1994). Results of conservative management of clinically localized prostate cancer.. N Engl J Med.

[OCR_00751] Chomczynski P., Sacchi N. (1987). Single-step method of RNA isolation by acid guanidinium thiocyanate-phenol-chloroform extraction.. Anal Biochem.

[OCR_00756] ENGELL H. C. (1955). Cancer cells in the circulating blood; a clinical study on the occurrence of cancer cells in the peripheral blood and in venous blood draining the tumour area at operation.. Acta Chir Scand Suppl.

[OCR_00760] Epstein J. I., Carmichael M. J., Pizov G., Walsh P. C. (1993). Influence of capsular penetration on progression following radical prostatectomy: a study of 196 cases with long-term followup.. J Urol.

[OCR_00773] Fidler I. J., Kripke M. L. (1977). Metastasis results from preexisting variant cells within a malignant tumor.. Science.

[OCR_00768] Fidler I. J. (1970). Metastasis: quantitative analysis of distribution and fate of tumor emboli labeled with 125 I-5-iodo-2'-deoxyuridine.. J Natl Cancer Inst.

[OCR_00779] Fleming C., Wasson J. H., Albertsen P. C., Barry M. J., Wennberg J. E. (1993). A decision analysis of alternative treatment strategies for clinically localized prostate cancer. Prostate Patient Outcomes Research Team.. JAMA.

[OCR_00783] Hamdy F. C., Lawry J., Anderson J. B., Parsons M. A., Rees R. C., Williams J. L. (1992). Circulating prostate specific antigen-positive cells correlate with metastatic prostate cancer.. Br J Urol.

[OCR_00792] Israeli R. S., Miller W. H., Su S. L., Powell C. T., Fair W. R., Samadi D. S., Huryk R. F., DeBlasio A., Edwards E. T., Wise G. J. (1994). Sensitive nested reverse transcription polymerase chain reaction detection of circulating prostatic tumor cells: comparison of prostate-specific membrane antigen and prostate-specific antigen-based assays.. Cancer Res.

[OCR_00797] Israeli R. S., Miller W. H., Su S. L., Samadi D. S., Powell C. T., Heston W. D., Wise G. J., Fair W. R. (1995). Sensitive detection of prostatic hematogenous tumor cell dissemination using prostate specific antigen and prostate specific membrane-derived primers in the polymerase chain reaction.. J Urol.

[OCR_00807] Katz A. E., Olsson C. A., Raffo A. J., Cama C., Perlman H., Seaman E., O'Toole K. M., McMahon D., Benson M. C., Buttyan R. (1994). Molecular staging of prostate cancer with the use of an enhanced reverse transcriptase-PCR assay.. Urology.

[OCR_00811] Lu-Yao G. L., McLerran D., Wasson J., Wennberg J. E. (1993). An assessment of radical prostatectomy. Time trends, geographic variation, and outcomes. The Prostate Patient Outcomes Research Team.. JAMA.

[OCR_00815] Moreno J. G., Croce C. M., Fischer R., Monne M., Vihko P., Mulholland S. G., Gomella L. G. (1992). Detection of hematogenous micrometastasis in patients with prostate cancer.. Cancer Res.

[OCR_00826] Poste G., Fidler I. J. (1980). The pathogenesis of cancer metastasis.. Nature.

[OCR_00833] Riegman P. H., Vlietstra R. J., van der Korput J. A., Romijn J. C., Trapman J. (1989). Characterization of the prostate-specific antigen gene: a novel human kallikrein-like gene.. Biochem Biophys Res Commun.

[OCR_00836] Schwartz R., Walk A., Toomes H., Schirrmacher V. (1985). Assay for the determination of human carcinoma cells in circulating blood.. J Cancer Res Clin Oncol.

[OCR_00841] Smith M. R., Biggar S., Hussain M. (1995). Prostate-specific antigen messenger RNA is expressed in non-prostate cells: implications for detection of micrometastases.. Cancer Res.

[OCR_00847] Warren E. R. (1936). MOUNTAIN ROAD CASUALTIES AMONG ANIMALS IN COLORADO.. Science.

